# Sandwich ELISA-Based Electrochemical Biosensor for Leptin in Control and Diet-Induced Obesity Mouse Model

**DOI:** 10.3390/bios11010007

**Published:** 2020-12-24

**Authors:** Ryong Sung, Yun Seok Heo

**Affiliations:** 1 Obesity-Mediated Disease Research (ODR) Center, School of Medicine, Keimyung University, Daegu 42601, Korea; ryong_s@kmu.ac.kr; 2 Department of Biomedical Engineering, School of Medicine, Keimyung University, Daegu 42601, Korea

**Keywords:** leptin analysis, electrochemical immunoassay, diet-induced obesity

## Abstract

Leptin is a peptide hormone produced primarily in adipose tissues. Leptin is considered a biomarker associated with obesity and obesity-mediated diseases. Biosensor detection of leptin in the blood may play a critical role as an indicator of dynamic pathological changes. In this paper, we introduce an electrochemical biosensor that adopts o-Phenylenediamine (oPD) on screen-printed gold electrodes (SPGEs) for detecting the leptin from a mouse model of diet-induced obesity (DIO). A linear calibration curve for the leptin concentration was obtained in the ranges from 0.1 to 20 ng/mL with a lower detection limit of 0.033 ng/mL. The leptin concentration was quantified with HRP (horseradish peroxidase)-catalyzed oxidation of oPD by two voltammetry methods: cyclic voltammetry (CV) and square-wave voltammetry (SWV). The proposed sandwich enzyme-linked immunosorbent assay (ELISA)-based electrochemical biosensor for the leptin in mouse blood serum showed high stability, sensitivity, selectivity, and effectivity compared to the commercial Leptin ELISA measurement. Thus, we believe that this leptin biosensor can be a sensitive analytical tool to detect low-levels of biomarkers in clinics and point-of-care testing (POCT).

## 1. Introduction

The number of overweight and obese people is becoming a significant burden on healthcare systems worldwide [1,2]. Obesity is associated with a high risk of physiological diseases and symptoms; hypertension, type 2 diabetes (T2D), sleep apnea, and various cancers [3,4,5,6,7]. As a 16-kDa peptide hormone, leptin is vital for regulating glucose metabolism, thermogenesis, and neuroendocrine axes. Blood leptin level is proportional to body fat amount, particularly white adipose tissues (WAT). It is reported that overweight and obese people have significantly higher blood leptin levels because of their larger WAT amounts [8,9,10]. The average concentration of blood leptin is approximately 10 ng/mL in adults with normal weight, whereas obese people have 30 ng/mL higher leptin levels [11]. The elevated leptin levels cause autoimmune, neurodegenerative, and cardiovascular diseases (CVD) [12,13,14,15]. Therefore, it is essential to measure leptin levels to inform healthcare management of obesity and obesity-mediated diseases.

In most practical fields, radioimmunoassay (RIA) [16], enzyme-linked immunosorbent assay (ELISA) [17], and Western blotting [18] are typically used to measure leptin levels. These techniques are effective and well established, but they have several limitations, such as complexity, expensiveness, and time-consuming procedures. Furthermore, they require sizeable instruments, skilled instrument technicians, and the use of complex protocols. Electrochemical techniques have shown advantages, such as high sensitivity, proper selectivity, low-cost, time-saving, and long-term stability in detecting various types of molecules [19]. Electrochemical biosensors have especially gained significant attention by measuring specific biomarkers (DNA, RNA, proteins, ions, and small molecules) in clinical and commercial fields [20,21]. ELISA is one of the most effective and commonly used methods for quantitating specific biomarkers with excellent performance; stability, sensitivity, selectivity, and effectivity [22]. Thus, it is crucial to develop a device combining both the benefits of electrochemical techniques and ELISA to measure the low biomarker concentrations in bodily fluids.

This article describes a sandwich ELISA-based electrochemical biosensor, using the strong binding affinity between the antigen and the antibody, enabling leptin’s detection in blood plasma samples at concentrations from 0.1 ng/mL to 20 ng/mL. We employed o-Phenylenediamine (oPD) as a powerful label substrate and developed a diet-induced obesity (DIO) mouse model fed by a high-fat content chow. It has been demonstrated that the DIO mouse models show physical and physiological similarities to obese humans [23,24]. It is also reasonable to use the DIO models as the representatives rather than genetically-derived models (*ob*/*ob* and *db*/*db* mice) because most obese human patients have non-engineered genes [25]. In addition, to the best of our knowledge, no one has reported the blood leptin level using an electrochemical biosensor together with oPD substrate and a DIO model mouse.

We successfully evaluated our proposed biosensor using various criteria, such as stability, sensitivity, selectivity, and effectivity. The biosensor also detected a meaningful difference in blood leptin level between normal mice and DIO model mice. Our findings suggested that the ELISA-based sandwich biosensor showed promise for the diagnosis of obesity and obesity-related diseases and opens a new window for the future development of commercially successful point-of-caring testing (POCT) products or devices.

## 2. Materials and Methods

### 2.1. Apparatus and Electrodes

All electrochemical measurements were performed using a CHI760E electrochemical workstation from CH Instruments (Austin, TX, USA). The screen-printed gold electrodes, SPGEs (DRP-C220AT), were purchased from Dropsens. These are composed of (i) a gold working electrode (diameter = 4 mm), (ii) a gold counter electrode, and (iii) a silver reference electrode. A cable connector linked the SPGEs to the workstation.

### 2.2. Reagents

All chemicals and reagents were of an analytical grade and used without any further purification steps. Phosphate buffered saline (PBS), Triton X-100, o-Phenylenediamine dihydrochloride (oPD), bovine serum albumin (BSA), potassium hexacyanoferrate III (K_3_[Fe(CN)_6_]), and potassium hexacyanoferrate II (K_4_[Fe(CN)_6_]) trihydrate were purchased from Sigma–Aldrich (St. Louis. MO, USA). 3,3′-dithiobis (sulfosuccinimidyl propionate) (DTSSP) was purchased from ThermoFisher. Streptavidin-HRP (horseradish peroxidase) solution was purchased from Abcam. Mouse leptin ELISA kits were obtained from Merck Millipore. Mouse leptin recombinant protein, mouse monoclonal anti-leptin antibody (as the capture antibody), and mouse polyclonal anti-leptin antibody (biotin-conjugated, as the detection antibody) were purchased from Enzo Life Sciences. All stock and buffer solutions were prepared using autoclaved Milli-Q ultra-filtered distilled and deionized water (18.2 MΩ-cm). All experiments were conducted at room temperature (23 ± 1 °C).

### 2.3. Diet-Induced Obesity Mice Model

All the animal experimental procedures and protocols were approved by the Keimyung University School of Medicine Animal Care and Use Committee (KM-2019-18R2). Male C57BL/6J mice (4 weeks old) were provided from Hyochang science (Daegu, Korea) and housed in standard cages in a 12-h light/dark cycle at 23 °C ± 1 °C with 50% ± 5% relative humidity. Before diet modification, the mice adapted for a week with normal diet chow. All mice were divided into two groups (n = 5 per group) with matched weights; high-fat diet (HFD) and normal diet (ND) mice. HFD mice were fed high-fat chow (60% kcal from fat), and the ND group fed normal chow (15% kcal from fat). Both groups were fed ad libitum for 12 weeks, and their body weight was measured daily. Blood was collected by cardiac puncture after 15 h of fasting with a K3 EDTA-coated tube and centrifuged at 4 °C. Plasma was separated and stored at −80 °C until analysis. Organs and tissues (liver, epididymal and subcutaneous fat) were collected after blood collection and weighed.

### 2.4. Leptin Biosensor Modification (SPGE/DTSSP/a-Leptin/Leptin/Biotinylated a-Leptin/Streptavidin-HRP/oPD)

**Scheme 1:**
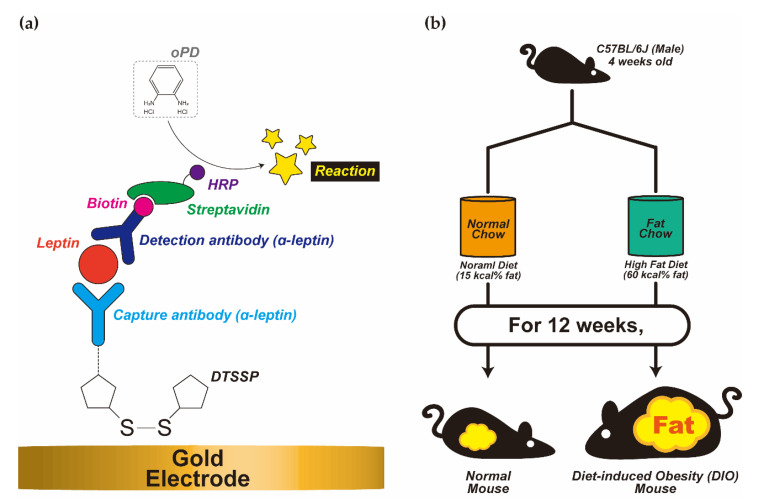
Schematic illustration of (**a**) a sandwich enzyme-linked immunosorbent assay (ELISA)-based electrochemical biosensor on screen-printed gold electrodes (SPGE) and (**b**) a procedure of diet-induced obesity (DIO) model development using C57BL/6J male mice.

A schematic illustration of a sandwich ELISA-based electrochemical biosensor is shown in Scheme 1a. Before preparing the biosensor, SPGEs were stored in pure ethanol overnight at 4 °C to remove impurities and were gently dried with a flow of Nitrogen gas. Subsequently, a 50 μL droplet of 5 mM DTSSP solution was applied to the electrode (working area only) and incubated for 2 h. A 50 μL droplet of anti-leptin antibody solution (10 μg/mL) was then applied as a capture antibody on the electrode for 1 h. To prevent unspecific binding, we applied a 50 μL droplet of a blocking buffer (1% BSA, 1% Triton X-100) to the electrode for 1 h, then gently rinsed it with the blocking buffer. A 50 μL droplet of the antigen solutions (leptin solutions or mouse blood plasma) was then applied onto the electrode and incubated at 4 °C in a humid chamber. In addition, a 50 μL droplet of a biotin-conjugated anti-leptin antibody solution (10 μg/mL) was applied on the electrode at room temperature for 1 h. A streptavidin-HRP solution (0.1 μg/mL) was then applied to the electrode at room temperature for 0.5 h. To complete the ELISA-based electrochemical biosensor’s preparation to detect leptin, a 50 μL droplet of oPD solution was applied as an electron mediator on the modified electrode and then incubated at room temperature for 0.5 h.

### 2.5. Electrochemical Measurement

The modified biosensor with multiple layers of SPGE/DTSSP/leptin, Ab./leptin/biotinylated leptin, and Ab./Strep-HRP/oPD was evaluated by two electrochemical detection methods; cyclic voltammetry (CV) and square-wave voltammetry (SWV). During CV, cyclic voltammograms were recorded at electrical potentials ranging from −0.5 V to 0.7 V with a scan rate of 100 mV/s. For SWV, the square wave voltammograms were recorded at electrical potentials ranging from −0.8 V to 0.0 V with a frequency of 15 Hz and an amplitude of 0.025 V. All experiments were performed and analyzed in triplicate at room temperature.

### 2.6. Statistics

All statistical analyses were performed using the Microsoft Excel 2016 (Microsoft Corp., Redmond, WA, USA) software. The level of statistical significance was set at *p* ≤ 0.001.

## 3. Results and Discussion

### 3.1. Diet-Induced Obesity Mice Model

**Figure 1:**
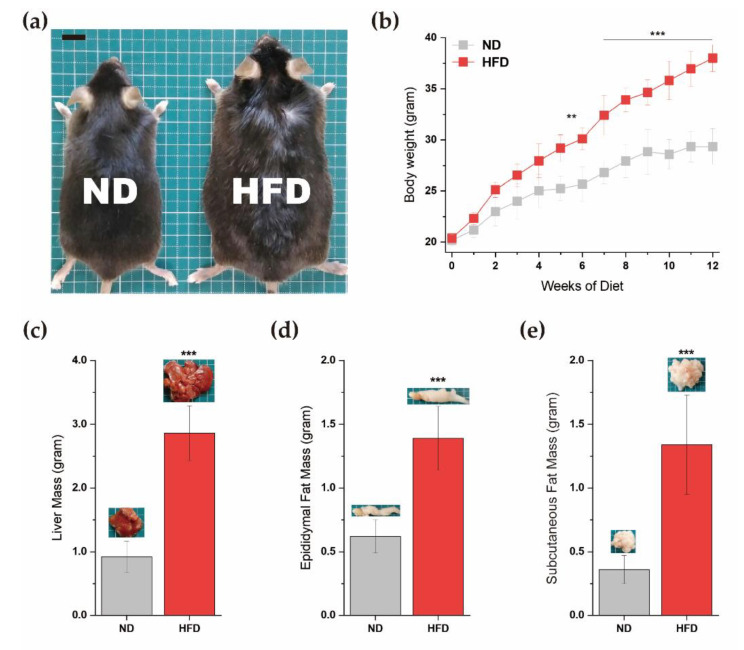
Comparison of the characteristics of the normal diet (ND) and high-fat diet (HFD) mouse groups (n = 5 per group). All measurements for the four characteristic parameters were performed after 12 weeks of diet exposure. (**a**) The appearances of representative ND and HFD mice (Scale bar = 1 cm) and (**b**) a chart of the time course of changes in body weight in each group, (**c**) liver mass (gram) in each group, (**d**) epididymal fat mass (gram) in each group, and (**e**) subcutaneous fat mass (gram) in each group (** *p* ≤ 0.01, *** *p* ≤ 0.001). (**c**–e) include representative images of the liver, epididymal fat, and subcutaneous fat, respectively. All static data are presented as mean ± standard deviation.

DIO model development using male C57BL/6J mice is shown in Scheme 1b. Male mice were chosen because, compared to the female mice, they are more vulnerable to HFD effects, such as weight gains, etc. [26]. In Figure 1, we summarized the characteristic comparisons of the normal diet (ND) mouse group and the high-fat diet (HFD) mouse group (n = 5 per each group). After 12 weeks of the diet treatments, we demonstrated four significant physical differences (body weight, liver mass, epididymal fat mass, and subcutaneous fat mass) between the two groups. The HFD mice had statistically increased in body weight (21.33 ± 2.48 g in ND mice and 29.96 ± 3.33 g in HFD mice) while the starting weights in each group were similar to each other (18.25 ± 0.25 g in ND mice and 18.49 ± 0.18 g in HFD mice). In terms of body weight, an increase in weight was detected after 2 weeks and became more apparent after 5 weeks of the diet. There was a significant weight difference after 10 weeks of the diet (21.68%) (Figure 1b). Liver mass was 0.92 g ± 0.25 g in ND and 2.86 g ± 0.43 g in HFD (Figure 1c). The liver weight was also dramatically increased in the HFD mice. Epididymal fat mass and subcutaneous fat mass were 0.62 g ± 0.13 g and 1.39 g ± 0.25 g, respectively. (Figure 1d,e). Both epididymal fat and subcutaneous fat are categorized as white adipose tissue (WAT). Thus, as shown in Figure 1d,e, the increases in both epididymal and subcutaneous fat mass mean increased total white adipose tissue (WAT). The most apparent difference between normal and obese groups was the body-fat ratio, especially between the brown adipose tissue (BAT) and white adipose tissue (WAT) [27]. The development of obesity mainly depends on the amount of WAT because WAT has the highest level of fat-containing adipocytes in mammals. Based on all changes in physical values, we determined that the DIO mice model was successfully established.

### 3.2. Stability

**Figure 2:**
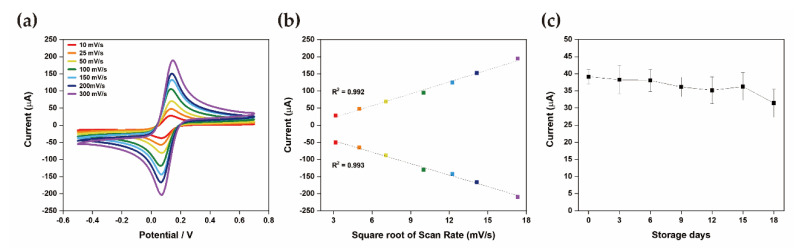
(**a**) Cyclic voltammetry (CV) response test for stability with 10 to 300 mV/s scan rates. (**b**) The oxidation and reduction current peaks from graph (**a**). (**c**) The long−term storage ability test in humid conditions at 4 °C over 18 days. Measurements were taken every 3 days and calculated as the mean value of peak current from 1.0 ng/mL leptin in triplicate.

We quantified the stability and long-term storage ability of the biosensor. First, we investigated the stability of the bare screen-printed gold electrodes by cyclic voltammetry (CV) at a range of scan rates. The CV with 10 to 300 mV/s scan rates was applied by using 0.1 M KCl solution with 5 mM potassium ferricyanide (III) solution. As shown in Figure 2a, the absolute values of anodic and cathodic peak currents showed a linear relationship with the square root of the scan rates. The correlation coefficients were 0.992 and 0.993 for the oxidation and reduction peaks, respectively (Figure 2b). These results demonstrated that the electrochemical reaction on the surface of SPGE was a diffusion-controlled process.

**Figure 3:**
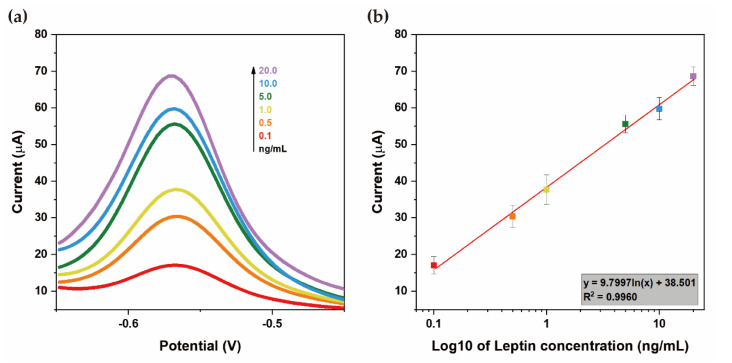
(**a**) Square−wave voltammetry (SWV) response curves of the biosensor toward various concentrations of leptin solutions from 0.1 to 20 ng/mL and (**b**) calibration plots displaying a robust linear relationship between the SWV peak currents and the logarithm of leptin concentrations. Each point was obtained and integrated from the peak currents in triplicate.

Additionally, we investigated the biosensor’s long-term storage ability after being kept under blocking buffer (1% BSA with 0.1% Triton X-100 in 1X PBS solution) for up to 18 days at 4 °C. Each biosensor was constructed as far as applying the layer of a streptavidin-HRP and stored in a 15 mL conical tube with 2 mL of blocking buffer. Stability data are shown in Figure 3. Each point indicates the mean value of peak current from three 1.0 ng/mL leptin measurements every 3 days (Figure 2c). A total of twenty-one SPGEs were used for this storage ability testing. After 18 days of testing, the biosensor’s electrochemical response maintained almost 80% (80.29%) of its initial response measured on the first day (day 0). This small variation over 18 days indicated that the biosensor has an excellent storage ability in humid conditions.

### 3.3. Sensitivity

To investigate biosensor sensitivity, the quantitative detection of leptin was evaluated by SWV measurement against the increasing concentration of leptin solutions (0.1, 0.5, 1.0, 5.0, 10, and 20 ng/mL). The solutions were prepared with mouse leptin recombinant proteins with specific binding affinity with an α-leptin antibody. The SWV responses for the electrochemical behaviors of leptin are shown in Figure 3a. In Figure 3b, calibration plots displayed a robust linear relationship between the SWV peak currents and the logarithm of leptin concentrations (R^2^ = 0.9867). Each of the points was obtained and integrated from the peak currents in triplicate. The limit of detection (LOD) was determined to be 0.033 ng/mL through the 3σ rule. The linearity and low LOD indicated that the sandwich ELISA-based electrochemical biosensor could be applied accurately and with high sensitivity to quantify leptin concentrations spanning the physiological range for mammals.

### 3.4. Selectivity

We investigated the biosensor’s selectivity, the biosensor’s ability to discriminate a specific biomarker, such as leptin, in a mixture of bodily fluids, such as blood plasma. Table 1 summarizes leptin recovery rates among four combinations of insulin, glucose, and urea with leptin. Closer to 100% recovery rates indicate fewer false-positive signals caused by non-specific binding with other compounds. The compounds were dissolved with distilled water and prepared at a physiologically realistic concentration (5 ng/mL for each compound). The electrochemical leptin signals in the mixtures were compared to that of a 5 ng/mL leptin solution as a control. All leptin recovery rates were close to 100%. These rates demonstrated that the biosensor had high selectivity towards leptin and did not experience non-specific binding with other compounds.

### 3.5. Effectivity

We also investigated the biosensor’s effectivity, which is the ability to distinguish the leptin levels in non-diluted blood from the two groups, ND and HFD mice. Table 2 summarizes the leptin concentrations from the ND (3.08–5.86 ng/mL) and HFD groups (11.58–18.58 ng/mL) with the Sandwich ELISA-based Leptin biosensor. Next, we compared the leptin level obtained from the Sandwich ELISA-based Leptin biosensor to that from a microplate reader using a commercial ELISA kit. The quantification differences between the biosensor and ELISA kit were less than 10% across all samples (Table 2). The ability to distinguish between ND and HFD mice, and comparable performance to the commercial ELISA kit, showed that the modified biosensor with electrochemical strategy could be applied in the research of obesity and obesity-related diseases.

## 4. Conclusions

Here, we reported a sandwich ELISA-based electrochemical biosensor for detecting leptin in blood plasma from ND and HFD mice. The biosensor consists of multi-layers of a DTSSP, a capture antibody, a detection antibody, and a streptavidin-HRP on the surface of a screen-printed gold electrode. The quantification of the leptin concentration was accomplished with HRP-catalyzed oxidation of oPD by two voltammetry methods; cyclic voltammetry (CV) and square-wave voltammetry (SWV). The proposed sandwich ELISA-based electrochemical biosensor for detecting leptin in mouse blood serum was evaluated as having high stability, sensitivity, selectivity, and effectivity. The leptin concentrations obtained by the biosensor were distinguishable between ND and HFD groups. Significantly, the leptin level obtained from the biosensor was comparable to that from a microplate reader using a commercial ELISA kit. Thus, we believe that our biosensor is a promising tool for diagnosing diverse biomarkers for obesity and obese-related diseases and opens a new window for the development of commercially successful point-of-caring testing (POCT) devices for humans [28,29].

## Figures and Tables

**Table 1:** Cross-reactivity study indicating biosensor leptin recovery rates in combination with insulin, glucose, and urea.

Sample (ng/mL)	Added (ng/mL)	Found * (ng/mL)	Recovery (%)
Leptin	5.0 (Leptin)	4.69 ± 0.36	93.8
Leptin + Insulin	5.0 (Leptin) + 5.0 (Insulin)	5.40 ± 0.34	108
Leptin + Glucose	5.0 (Leptin) + 5.0 (Glucose)	4.96 ± 0.55	99.2
Leptin + Urea	5.0 (Leptin) + 5.0 (Urea)	4.71 ± 0.76	94.2
Leptin + Insulin + Glucose	5.0 (Leptin) + 5.0 (Insulin) + 5.0 (Glucose)	5.51 ± 0.61	110

* The experiments were performed in triplicate, with the results presented as mean ± standard deviation.

**Table 2:** The blood leptin levels of high-fat diet (HFD) and normal diet (ND) mice with a commercial Leptin enzyme-linked immunosorbent assay (ELISA) kit and the sandwich ELISA-based leptin biosensor.

Samples (Plasma)	Commercial Leptin ELISA Kit (Absorbance) (ng/mL)	Sandwich ELISA-Based Leptin Biosensor * (ng/mL)	Difference (%)
ND S1	04.25	04.64 ± 0.26	8.40
ND S2	05.27	05.02 ± 0.37	4.98
ND S3	03.21	03.08 ± 0.49	4.22
ND S4	03.11	03.42 ± 0.22	9.06
ND S5	06.18	05.86 ± 0.39	5.46
HFD S1	12.69	11.58 ± 0.41	9.59
HFD S2	17.25	18.58 ± 0.55	7.16
HFD S3	15.58	15.02 ± 0.49	3.73
HFD S4	11.08	12.20 ± 0.71	9.18
HFD S5	16.93	15.82 ± 0.65	7.02

* The experiments were performed in triplicate, with the results presented as mean ± standard deviation. (Here, S1 represents Sample number 1, other numbers follow in sequence).
